# Spatially targeted brain cancer immunotherapy with closed-loop controlled focused ultrasound and immune checkpoint blockade

**DOI:** 10.1126/sciadv.add2288

**Published:** 2022-11-18

**Authors:** Hohyun Lee, Yutong Guo, James L. Ross, Scott Schoen, F. Levent Degertekin, Costas Arvanitis

**Affiliations:** ^1^ G.W. School of Mechanical Engineering, Georgia Institute of Technology, Atlanta, GA, USA.; ^2^ Department of Microbiology and Immunology, Emory University, Atlanta, GA, USA.; ^3^ Emory Vaccine Center, Emory University School of Medicine, Atlanta, GA, USA.; ^4^ Harvard Medical School and Massachusetts General Hospital, Boston, MA, USA.; ^5^ Georgia Institute of Technology and Emory University, Department of Biomedical Engineering, Atlanta, GA, USA.

## Abstract

Despite the challenges in treating glioblastomas (GBMs) with immune adjuvants, increasing evidence suggests that targeting the immune cells within the tumor microenvironment (TME) can lead to improved responses. Here, we present a closed-loop controlled, microbubble-enhanced focused ultrasound (MB-FUS) system and test its abilities to safely and effectively treat GBMs using immune checkpoint blockade. The proposed system can fine-tune the exposure settings to promote MB acoustic emission–dependent expression of the proinflammatory marker ICAM-1 and delivery of anti-PD1 in a mouse model of GBM. In addition to enhanced interaction of proinflammatory macrophages within the PD1-expressing TME and significant improvement in survival (*P* < 0.05), the combined treatment induced long-lived memory T cell formation within the brain that supported tumor rejection in rechallenge experiments. Collectively, our findings demonstrate the ability of MB-FUS to augment the therapeutic impact of immune checkpoint blockade in GBMs and reinforce the notion of spatially tumor-targeted (loco-regional) brain cancer immunotherapy.

## Teaser

Closed-loop ultrasound augments the therapeutic impact of anti-PD1 in glioblastoma and induces long-lived memory T-cell formation.

## INTRODUCTION

The recent discovery of functional lymphatic vessels in the meninges, along with increasing evidence of T cell entry and immunosurveillance within the brain (*1*–*3*), created hope that immune adjuvants could be beneficial against brain tumors (*4*, *5*). While T cell–activating immunotherapies, such as anti-PD1, led to remarkable responses in brain metastases (*6*), the outcomes in the treatment of primary brain tumors, such as glioblastoma (GBM), were disappointing (*7*, *8*). Although the reason for these poor outcomes remains elusive, increasing evidence suggests that more effective targeting of the immune cells in the tumor microenvironment (TME) may lead to improved outcomes (*9*–*12*). In addition, intensive treatment protocols based on multiple and frequent dosing have shown improved outcomes (*10*), supporting the notion that improved local delivery can be beneficial. However, for immune adjuvants to have a direct biological impact on the GBM TME, they must reach the immune cells in the TME, which can make up to 30% of the GBM mass (*13*, *14*). While the vasculature in GBM is abnormally leaky [referred to as the blood-tumor barrier (BTB)], its permeability is highly heterogeneous, with significant fractions of it being similar to healthy brain vessels [referred to as blood-brain barrier (BBB)], which is impenetrable to the vast majority of therapeutic agents, including antibodies (*15*). Because of these characteristics, the BBB/BTB has been implicated as a limiting factor for the delivery of therapeutics (e.g., anti-PD1) and adequate antigen presentation (*5*, *16*). Hence, strategies to increase local delivery and penetration of immune adjuvants in the GBM TME can be critical for reprogramming immune cells within the TME. Such approaches may also improve antigen presentation and better control immune-related adverse events by supporting less aggressive protocols (*17*, *18*).

Low-intensity focused ultrasound (FUS) combined with microbubble (MB) ultrasound (vascular) contrast agents provide a physical method to reversibly increase the permeability of the BBB (*19*) and improve the uptake and penetration of anticancer agents in brain tumors (*20*). Following extensive preclinical research that demonstrated safe and effective delivery of a wide range of anticancer agents across different murine brain tumor models using MB-FUS (*21*), early clinical trials confirmed its ability to increase the BBB/BTB permeability in infiltrating gliomas (*22*) and enhance the accumulation of the antibody trastuzumab in HER2^+^ breast-brain metastases (*23*). Although MB-FUS has been primarily considered as a drug delivery technology, recent studies have shown that it can also invoke inflammatory responses in healthy mice brains in an MB dose– and FUS exposure–dependent manner (i.e., from nonsignificant or minimal to very strong responses) (*24*, *25*). Inspired by these observations, emerging investigations have demonstrated that MB-FUS can enhance the accumulation of immune adjuvants in the brain TME and improve survival in different murine brain tumor models, including GBM (*26*–*28*). Despite these encouraging findings and extended work on healthy brains and brain tumors, as well as other disease models (e.g., Alzheimer’s) (*25*, *29*–*32*), the treatment window (i.e., FUS exposure) to elicit distinct immuno-mechano-biological effects and promote effective therapeutic trafficking in the brain TME remains poorly defined. This is because current studies in brain tumors typically report only the estimated focal pressure (*26*–*28*, *32*) and not the MB acoustic emissions generated during the sonication (*29*, *33*, *34*), which is critical for making accurate inferences about the strength and type (stable versus inertial) of the MB oscillation. Moreover, current investigations offer limited insights on the penetration and uptake of immune adjuvants, such as anti-PD1, in the brain TME (*27*, *28*), which hinders our ability to fully assess the presumed advantages of the proposed strategy. Likewise, the assessment of immune cell trafficking and changes in their phenotype (e.g., M1 versus M2 tumor-associated macrophage/microglia polarization) in response to the combined treatment remains unexplored (*27*). As a result, the relationship among changes in the strength and type of MB oscillation, BBB/BTB phenotype, penetration and distribution of immune adjuvants, and their impact on immune cell trafficking and therapeutic efficacy in the brain TME is yet to be established (*35*), which hinders the effective integration of these technologies.

Our ability to elucidate the potential benefits of spatially targeted brain cancer immunotherapy enabled by MB-FUS and translate them to the clinic also hinges on our ability to control the cerebrovascular MB dynamics. For instance, in some investigations, the inflammatory responses observed in healthy brains along with the putative increase in cytotoxic immune cell accumulation have not been confirmed in brain tumors (*32*), suggesting that higher pressures might be required. However, higher pressures can lead to higher infiltration of CD4^+^ lymphocytes (*36*), presumably due to vessel/tissue mechanical damage linked to MB collapse (i.e., inertial cavitation). The former has been associated with unfavorable prognosis in GBM (*37*) and may thus dampen any potential therapeutic benefits emerging from this neurointervention. Controlling the MB oscillation in the brain is not trivial, as it is a highly nonlinear phenomenon that is prone to instabilities (i.e., bubble collapse) (*38*). Its behavior is also sensitive to ultrasound excitation parameters, MB properties and concentration (*39*), and microenvironmental conditions, such as proximity to interfaces (i.e., vessel wall) (*40*). To mitigate these challenges, methods that rely on the MB acoustic emission (AE) spectral content have been proposed to adjust the focal pressure and promote specific type and strength of oscillation (*21*). However, their performance in the context of brain cancer immunotherapy is yet to be determined (*35*). Moreover, current methods are not responsive to temporal changes in MB concentration (*41*) that can make the controllers diverge (i.e., increase the pressure as a function of time) (*42*). Brain vessels may also respond to MB vibrations with abrupt changes in blood flow (*43*, *44*) that may in turn transiently change both their local concentration and their interaction with the vessel wall. Attaining and sustaining the desirable MB oscillations for eliciting distinct immuno-mechano-biological effects (*24*, *25*) in this highly dynamic environment, where a shift from stable oscillation to violent collapse can occur within a few tens of kilopascals, is both important and challenging (*21*).

Here, we present a closed-loop controlled MB-enhanced focused US system that is based on MB AE and demonstrate its ability to monitor their strength and kinetics and fine-tune the exposure settings to attain and sustain desirable MB oscillation in healthy and tumor-bearing mice brains. Moreover, we hypothesize that closed-loop control of MB dynamics can promote distinct responses in BBB/BTB phenotype and facilitate safe and effective anti-PD1 delivery in the GBM TME. Assessment of the BBB/BTB physical and molecular properties, anti-PD1 penetration and uptake, and its impact on immune cell trafficking, along with monitoring of treatment response rates and durability are used to test our hypothesis and support the effective integration and translation to the clinics of brain cancer immunotherapy with MB-FUS and immune checkpoint blockade.

## RESULTS

### Closed-loop controlled MB-FUS system promotes distinct changes in the BBB phenotype

**Fig. 1.**
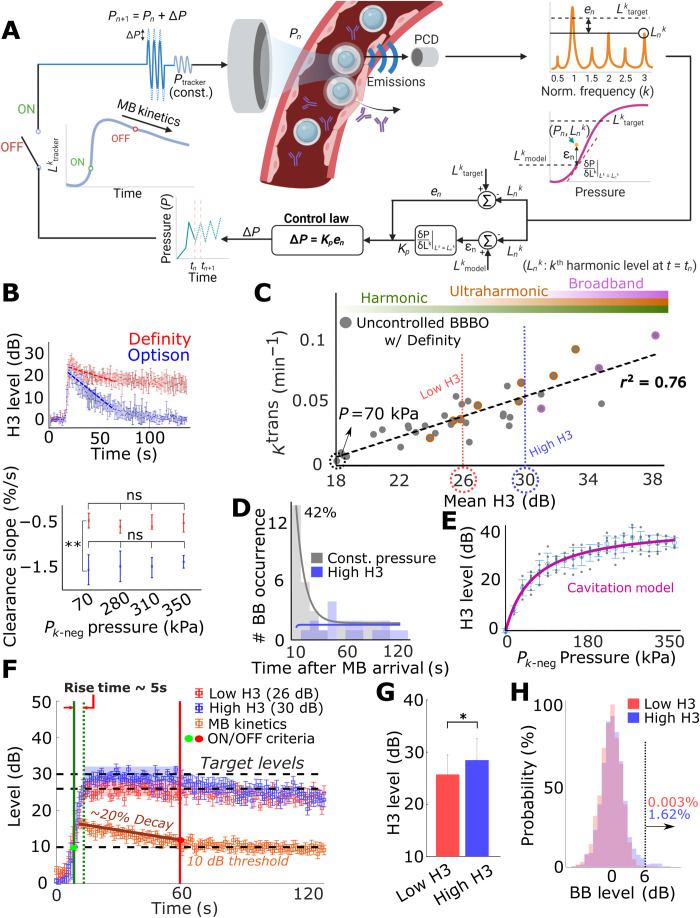
Design, training, and performance evaluation of the closed-loop controller. (**A**) Schematic showing the design of the controller. (**B**) Comparison of cerebrovascular clearance rate between Optison and Definity using the third harmonic (H3, after background removal). Optison showed a higher clearance rate (~1.5%/s) compared to Definity (~0.5%/s) (**P < 0.01). Clearance rates were independent of pressure. (**C**) Controller training. Mean H3 (during 40 s after MB arrival) showed a positive correlation (*r*^2^ = 0.76) with *K*^trans^. Two different target levels, low H3 (26 dB) and high H3 (30 dB), were selected from training data to represent different MB dynamics regime (weak-stable and strong-stable cavitation, see Methods for details). The ultraharmonic region (orange circles) indicates sonication with at least one ultraharmonic emission occurrence. The broadband region (pink circles) indicates sonication with more than 3% broadband emission occurrence. *P*_tracker_ was chosen at 70 kPa because it resulted in lowest *K*_trans_. (**D**) Broadband occurrence distribution during MB-FUS. Total broadband events (42.42%) were clustered at 10 s after MB arrival (gray). With the controller, no broadband emissions were detected during that time (blue). (**E**) Cavitation threshold model. AEs were recorded with pressure ranging from 0 to 350 kPa (*n* = 4). (**F** and **G**) Performance of closed-loop controller. A 5-s rise time and a 3.65-dB precision were achieved. Controller operation was governed by MB kinetics: 10-dB kinetic rise triggered the operation, and 20% kinetic decay ceased the operation. The selected target levels were distinguishable (*P < 0.05, low H3: *n* = 8 and high H3: *n* = 12). (**H**) Broadband-level distribution during controlled sonication. High H3 group had 1.62% of broadband (>6 dB) occurrence; low H3 group had 0.003% broadband occurrence. ns, not significant.

To control the cerebrovascular MB dynamics, we developed a closed-loop controlled MB-enhanced FUS system that uses specific frequency bands from the MB echoes (harmonic and ultra-harmonic for stable and broadband for inertial oscillation; fig. S1) to locally detect and control the MB dynamics (Fig. 1). The system is composed of a FUS transducer and a confocally and coaxially aligned imaging transducer that is mounted on a motorized three-dimensional (3D) positioning system. When in active mode (pulse echo), the imaging transducer is used for brain target identification (i.e., neuronavigation) based on imaging of the skull and fiducial markers placed in the eyes of the mice (fig. S2). Its targeting precision is 500 ± 140 μm. When operating in passive mode (listening only), it allows real-time monitoring of the MB AE during the sonication. The recorded AE is then used as input in a proportional feedback control law (P-control) that adjusts the FUS input to gradually reach and maintain a target AE level (“*L*_target_”). In its current implementation, the P-control applies a correction to the input (pressure) depending on the error between target and current harmonic emissions level (“*e_n_*”) and its gain (“*K_p_*”) to approach the target harmonic level gradually and steadily (Fig. 1A).

To start the controller, maximize its operation duration, and minimize divergence in response to temporal changes in the MB concentration, we incorporated a low and constant amplitude pulse to track the MB kinetics (temporal changes in AE signal) that interleaves the controlled input pulses. When a 10-dB increase in the third harmonic signal (H3) of this MB tracking pulse compared to background noise is detected (i.e., MB arrival to the brain), the controller is designed to start its operation. On the other hand, when there is a 20% decay in the H3 (third harmonic signal) level, compared to peak intensity, the controller ceases its operation and a constant input pressure is applied (i.e., the one used last by the controller) until the end of the sonication (≍2 min). In aggregate, the controller is designed to control MB dynamics with a closed-loop law, using an input-controlled pulse, while being responsive to MB kinetics using constant MB tracking pulse (Fig. 1A, see Methods for details).

To train the controller and assess its performance in vivo, we first identified the MB type to use by assessing the clearance rate of two U.S. Food and Drug Administration (FDA)–approved MBs, Definity (Lantheus Medical Imaging) and Optison (GE Healthcare), using constant pressure FUS pulses. Throughout the study, we used the Definity MBs, as it had considerably slower cerebrovascular MB clearance (0.5%/s) as compared to Optison MBs (1.5%/s) (Fig. 1B). The relatively slow clearance rate of Definity MBs allows the controller to operate for the longest possible duration and ensures more robust results. Second, we investigated the relationship between H3 (third harmonic) level, (i.e., controller output) and *K*^trans^ value, which is estimated using dynamic contrast-enhanced magnetic resonance imaging (DCE-MRI) and provides a measure of vessel permeability (assuming constant perfusion). We found that the AE and *K*^trans^ values were highly correlated (*R*^2^ = 0.76; Fig. 1C), underscoring the potential to control the changes in the BBB permeability using the MB AE and, in particular, H3 level. During these uncontrolled (constant pressure sonication, ranging from 70 to 350 kPa for 2 min) training sessions, we observed that 42% of the total broadband emission occurrences were present during the initial 10 s after the MB arrival to the brain (Fig. 1D, gray). These observations indicate that the MB concentration, which is highest at that time point, can affect their dynamics and reinforce the notion that control methods can be crucial during abrupt changes in MB concentration.

While these data are essential for training the controller, they are also very useful for selecting the pressure level for the MB tracking pulse (i.e., *K*^trans^ value equal to baseline, 70 kPa) and identifying distinct target AE levels to assess the performance of the controller. Last, we determined the model for the controller algorithm by rapidly sonicating a target region with pressure ranging from 0 to 350 kPa (Fig. 1E) and fitting a hypertangent model onto pressure-AE (H3) relationship (Fig. 1A, magenta curve; for details of using this model, see Methods). While the current cavitation threshold model is obtained from one mouse, the controller can undergo consistent learning process via optimization of this model using accumulated data (i.e., input pressure and output AE) from future sonications (fig. S3).

To assess the performance of the controller, we set the target AE at two close but distinct levels, 26 dB (low H3) and 30 dB (high H3) (see Fig. 1C), and sonicated the left and right brain hemispheres of mice, respectively. As designed, the controller’s operation started when MB arrival was detected (10 dB) and ceased when MB concentration decayed (20% reduction in the decibel level). This led to an average controller operation time of 41.65 ± 14.85 s. During this period, the controller was able to reach these two distinct target levels with mean rise time of 5 s and precision of ±3.65 dB (Fig. 1, F and G). Although these data demonstrate the controller’s ability to fine-tune the sonication pressure and attain the desired target MB AE level, the controller performance for the proposed application is ultimately assessed by its combined ability to suppress broadband emissions while promoting desirable changes in the BBB phenotype. To suppress broadband emissions throughout the entire sonication, the controller takes a maximum negative step when broadband signal is detected. With this functionality, which is active even when the controller is not active (fig. S4), broadband emissions (6 dB above the background noise) during all sonications (5 mice, 20 targets) occurred during fewer than 0.4 and 2% of events at low and high H3 target levels, respectively (Fig. 1H). The high occurrence of broadband emissions during MB arrival to the brain that was observed in controller training was effectively suppressed (Fig. 1D, blue), further supporting the abilities of the proposed controller to respond and correct the MB dynamics when abrupt changes in MB concentration occur. Without these safety features, significant broadband emission and tissue damage can occur (fig. S5). Note that because of this safety feature, the high H3 level appears below the threshold value of 30 dB (Fig. 1F).

**Fig. 2.**
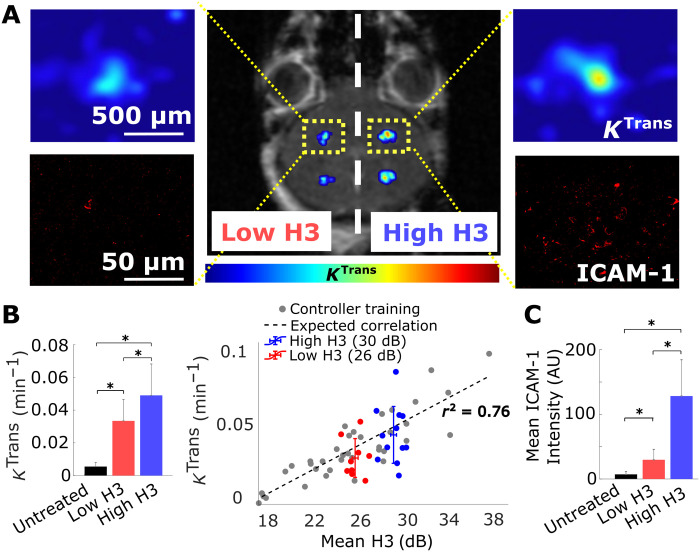
Closed-loop controlled BBB opening in healthy brain. (**A**) Representative image showing enhancements of *K*^trans^ (color bar represents pharmacokinetic map intensity) and ICAM-1 in healthy brain after closed-loop controlled sonication with two target AE levels. (**B**) Quantification of *K*^trans^ after sonication. Permeability changes after the sonication at different target levels had a significant increasing trend and correlated well with the training data (*n* = 5 animals total, two sonications of each target level at both sides for four animals, and one animal dedicated only for four targets of high H3, *P < 0.05). Untreated represents the untreated contralateral hemisphere. (**C**) Quantification of ICAM-1 expression at sonicated regions. ICAM-1 had exposure-dependent up-regulation that follows the *K*^trans^ trend (*P < 0.05). Untreated represents the untreated contralateral hemisphere. One-way analysis of variance (ANOVA) was used as significant test; error bars indicate SE. AU, arbitrary units.

To assess the abilities of the controller to tune BBB permeability, we collected DCE-MRI immediately after the sonications (low and high H3) (Fig. 2A). Our analysis shows that in healthy brain, the *K*^trans^ values (Fig. 2B) were at the expected target level. Moreover, at 6 hours after sonication, we assessed the expression of ICAM-1 (Fig. 2A), as several studies in the past have indicated that its up-regulation correlates well with broader immuno-mechano-biological responses in the brain following MB-FUS (*24*, *25*, *45*). Quantitative analysis of the observed ICAM-1 expression in the immunofluorescent microscopy datasets indicated that similar to *K*^trans^, the expression of ICAM-1 (Fig. 2C) scales proportionally to the AE target level. Together, these data demonstrate the ability of the proposed controller to fine-tune the MB AE and support our hypothesis that our control methods can be used to noninvasively promote distinct changes in the BBB phenotype.

### The closed-loop controlled MB-FUS system is robust to biological variability introduced by brain tumors

**Fig. 3.**
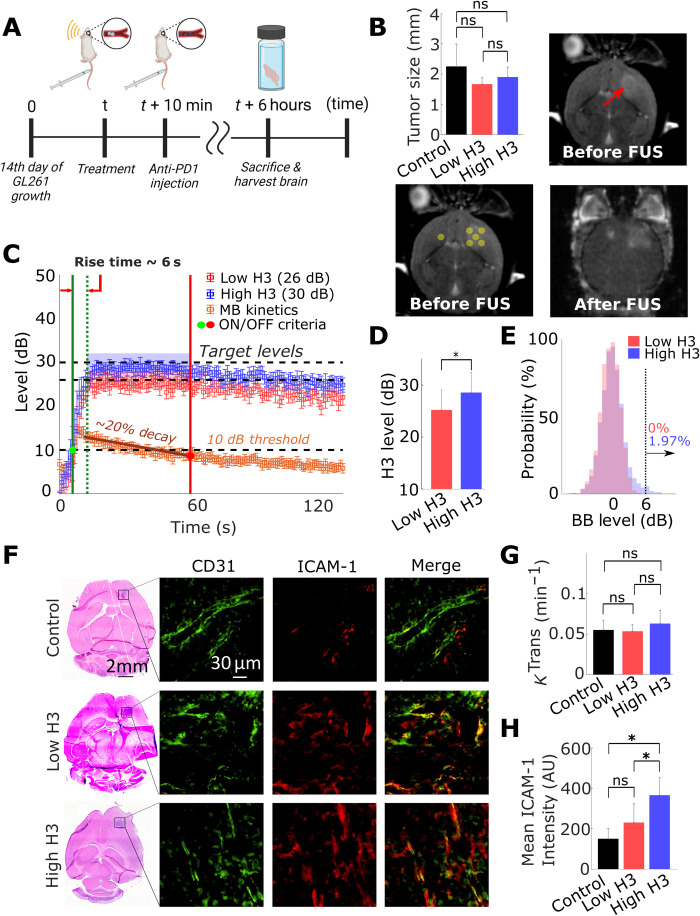
Closed-loop control of ICAM-1 up-regulation in TME. (**A**) Experimental protocol used in tumor-bearing mice. (**B**) Sonication pattern and tumor size quantification. Same target levels for closed-loop controller from healthy mice study were used (26 and 30 dB). Bottom left: Sonication pattern for each target level group (control, *n* = 4; low H3, *n* = 3; and high H3, *n* = 5) is shown/overlaid in the T1-weighted MRI with circles. One healthy brain target was sonicated to confirm the targeting accuracy of USgFUS system. Control group had anti-PD1 injection without sonication. Top left: Quantification of tumor sizes across groups. Tumor diameter across all groups was approximately equal to 2 mm. (**C**) Closed-loop controller performance during sonication. Controller achieved mean rise time of 6 s with precision of 3 dB. (**D**) Performance evaluation of the controller in the brain TME. The controller was able to distinguish two target AE levels (*P < 0.05). (**E**) Broadband emission probability during controlled sonication. High H3 (30 dB) group had 1.97% of broadband (>6 dB) occurrence; low H3 (26 dB) group had no broadband emission. (**F**) Representative H&E and fluorescence microscopy images showing ICAM-1 up-regulation in the high H3 group. (**G**) Quantification of *K*^trans^ in tumors. *K*^trans^ value did not have significant variance across the groups (*P > 0.05). (**H**) Quantification of ICAM-1 expression in tumors. ICAM-1 expression increased significantly (*P < 0.05) at high H3 target level compared to control group and low H3 group. One-way ANOVA was used as significant test; error bars indicate SE.

While the previous investigations were critical for tuning and assessing the performance of the proposed closed-loop controller in healthy mice and diseases that do not affect the BBB phenotype, it is unclear whether the observed responses and reported correlations will be preserved in the brain TME. The latter is characterized by high biological heterogeneity and distinct physical traits (*46*) that may affect both the MB dynamics and kinetics and their relative impact on the TME. To assess the performance of the controller and its ability to promote controlled changes on BBB/BTB phenotype in the brain TME, we used the murine GL261 glioma tumor model and the same AE levels we used in healthy mice. To minimize differences in the BBB/BTB permeability and other tumor microenvironmental factors across different animals that can be influenced by tumor size, mice were distributed equally between groups based on tumor size, as indicated by MRI, and sonicated at five nonoverlapping regions to cover the entire tumor and its margin (Fig. 3, A and B). The controller rise time (6 s) and precision (3 dB) (Fig. 3C) along with the AE and exposure level between low and high H3 groups (Fig. 3D) were similar to the one observed in healthy mice. We also observed less than 2% of broadband emissions throughout the high H3 sonications and none in the low H3 sonication (Fig. 3E). DCE-MRI showed no difference in the BBB/BTB permeability across all groups, although fluorescence microscopy indicated similar increasing trends in ICAM-1 expression in the brain TME with healthy brains (Fig. 3, F to H). This observation is contrary to current understanding from investigations in healthy brains, where changes in BBB permeability, as evidenced by DCE-MRI, are considered a good predictor of MB-FUS–mediated inflammatory responses (i.e., up-regulation in the expression of ICAM-1) (*47*). Together, our findings demonstrate that the controller is robust to biological variability introduced by the GL261 tumors and that the level of the AE is a better predictor of MB-FUS–mediated changes in the BBB/BTB phenotype, evidenced by up-regulation in ICAM-1 expression, as compared to *K*^trans^ values obtained by DCE-MRI.

### Closed-loop controlled MB-FUS promotes targeted anti-PD1 delivery to GL261 tumor tissue and enhances the interaction of macrophages with the brain TME

**Fig. 4.**
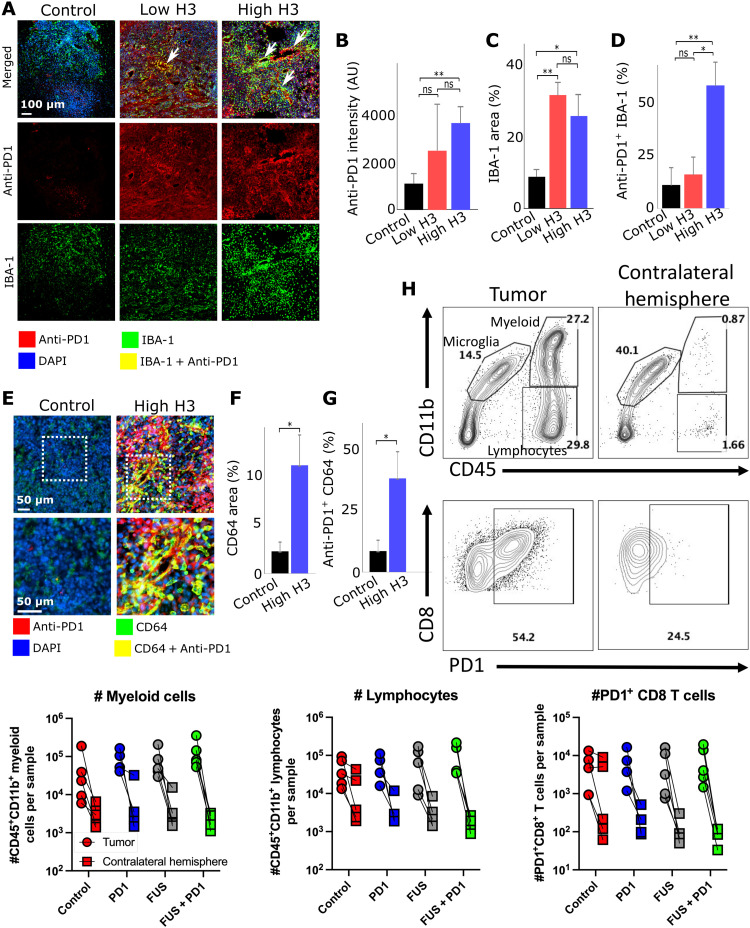
Closed-loop control of anti-PD1 delivery and resulting macrophage accumulation in TME. (**A**) Representative fluorescent microscopy of anti-PD1 delivery and TAM quantification (control, *n* = 4; low H3, *n* = 3; and high H3, *n* = 5). (**B**) Quantification of anti-PD1 delivery in tumor. High H3 group had 2.5-fold improvement in anti-PD1 intensity in TME compared to control group (**P < 0.01). No significances were observed between low H3 and control group, as well as high H3 group. (**C**) Quantification of IBA-1 area in TME. High H3 group and low H3 group had significant increase (3- and 3.5-fold) in IBA-1 area (*P < 0.05 and **P < 0.01). No significances were observed between high H3 and low H3 group. (**D**) Quantification of anti-PD1/IBA-1 colocalization. Percentages indicate colocalized area over IBA-1^+^ area. High H3 group had significant increase in colocalization area (5.5-fold) compared to control group (**P < 0.01) and a 3.5-fold increase compared to low H3 group (*P < 0.05). No significant colocalization was observed between control and low H3 group. (**E**) Representative fluorescent microscopy of anti-PD1 delivery and CD64. Red, green, blue, and yellow indicate anti-PD1^+^, CD64^+^, DAPI, and anti-PD1/CD64 colocalization, respectively. (**F**) Quantification of CD64^+^ area. High H3 group had a significant fivefold (*P < 0.05) higher expression of CD64^+^ area compared to control group. (**G**) Quantification of CD64/anti-PD1 colocalization. Percentages indicate colocalized area over CD64^+^ area. High H3 group had a fivefold increase in colocalization compared to control group (*P < 0.05). (**H**) PD1-stained flow cytometry (anti-PD1 only, *n* = 4; for all other groups, *n* = 5). More channels can be seen in fig. S7. One-way ANOVA was used as significant test; error bars indicate SE

In addition to observed changes in the BBB/BTB phenotype, it is important to assess the abilities of the proposed system to increase the local delivery and penetration of immune checkpoint inhibitors and affect the interaction of immune cells with the GBM TME. This is because vessel permeability is size dependent [i.e., very high for the low–molecular weight MR contrast agent (<1 kDa) and significantly lower for the much larger anti-PD1 antibody (≍150 kDa)] and DCE MRI (*K*^trans^) is not able to capture it. Thus, first, we assessed whether the proposed closed-loop controlled MB-FUS system can improve the delivery of anti-PD1 in GL261 gliomas. Immunofluorescence staining showed an increasing trend in anti-PD1 delivery as a function of target AE level (Fig. 4, A and B). We found that at the high H3 group, the anti-PD1 intensity was 2.5-fold higher as compared to the unsonicated control group (*P* < 0.01). Beyond the improved delivery, we also observed better penetration and distribution of the anti-PD1 in the H3 group as compared to control, which is characterized by high variability in the distribution of anti-PD1 with several tumor regions (i.e., which were positive to IBA-1 staining, but characterized by low to no anti-PD1 delivery) (Fig. 4A).

Considering the increasing evidence that anti-PD1 can support the development of an antitumorigenic M1 phenotype and/or increase the phagocytic capacity of tumor-associated macrophages/microglia (TAMs) (*9*, *10*), which are distributed in and around the tumor and can make up to 30% of the GBM mass (*13*, *14*), we analyzed the distribution and proximity of anti-PD1 and macrophages/microglia in the TME. When staining for IBA-1, which stains for macrophages and microglia (*48*), we found that the IBA-1^+^ tumor area had increased by 3.5-fold (*P* < 0.01) and 3-fold (*P* < 0.05) at 6 hours after the sonications with low and high H3, respectively, as compared to the control nonsonicated group (Fig. 4C). Our analysis indicated that the colocalization of macrophages/microglia and anti-PD1 was 5.5-fold higher between the control group and high H3 group (*P* < 0.01; Fig. 4D). We also observed a substantial number of IBA-1^+^ cells in close proximity to vasculature, suggesting that the application of MB-FUS augmented their interaction with the brain vessels and BBB/BTB. To assess the phenotype of the macrophages and microglia, we stained for CD64, which is overexpressed in macrophages that tend to display proinflammatory characteristics (M1 polarization) (*49*). Notably, in the group with the most noticeable anti-PD1 delivery (high H3 group; Fig. 4E), we observed a fivefold increase in the tumor area covered by CD64^+^ cells (Fig. 4F) and a fivefold increase in the colocalization between CD64 and anti-PD1, as compared to control (Fig. 4G).

Last, we assessed the presence of PD1^+^ CD8 T cells within the GBM TME, as well as a broad panel of other immune cells with flow cytometry 12 hours after sonication (figs. S6 and S7). We did not find any inflammatory differences among treatment groups in tumor tissue based on the number of CD4^+^ or CD8^+^ T cells, B cells, neutrophils, dendritic cells, and myeloid cells; however, our analysis showed an abundance of PD1^+^ CD8 T cells within the TME that was not observed in the contralateral nontumor hemisphere that was sonicated with the same exposure conditions (high H3) (Fig. 4H). This observation was also applicable to each immune cell population analyzed, providing further evidence for the safety and specificity of the proposed controller, as it is desirable to prevent deleterious treatment-induced inflammation in healthy brain tissue while simultaneously targeting PD1^+^ immune cells in the TME. Furthermore, there was a notable presence of PD1^+^TCF1^+^CD8 T cells in the tumor (fig. S7), which serve as resource cells for anti-PD1 therapy (*50*, *51*), suggesting that enhanced delivery of PD1 treatment antibody may lead to a therapeutic response. In aggregate, our findings demonstrate that the proposed closed-loop controlled MB-FUS system significantly improved the delivery of anti-PD1 in the GL261 brain TME, promoted a proinflammatory microenvironment, and had minimal inflammatory side effects in healthy brain tissue.

### Closed-loop controlled MB-FUS in combination with anti-PD1 promotes antitumor immunity in GL261 tumors

**Fig. 5.**
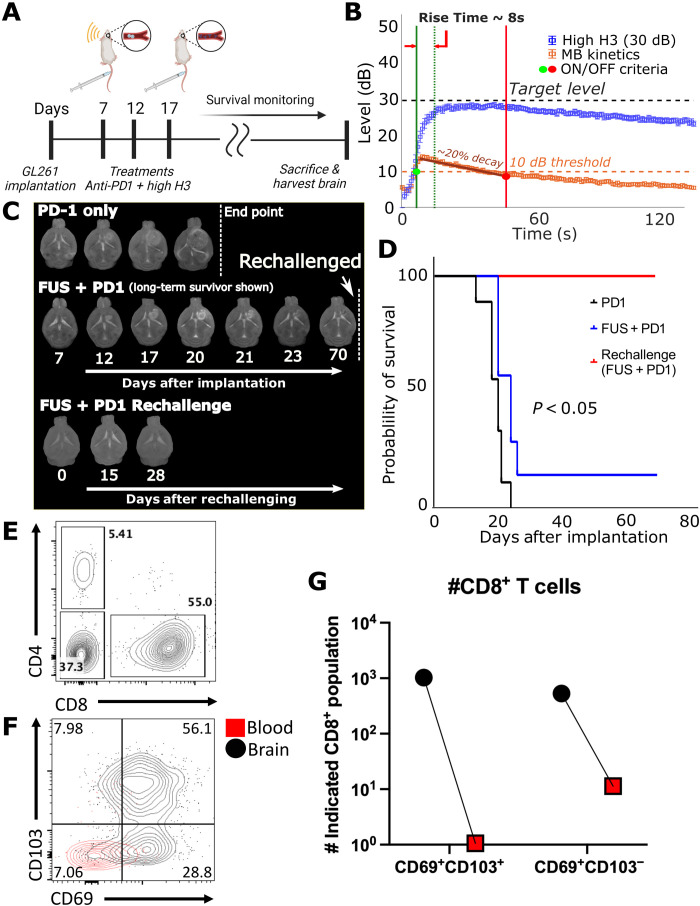
Survival study. (**A**) Experimental protocol of survival study (control, *n* = 9; high H3, *n* = 9). (**B**) Acoustic emissions for all survival mice. (**C**) T2-weighted MR images throughout treatments and survival monitoring. One animal (shown as FUS + PD1) survived and had its tumor cured and was rechallenged at day 70. Rechallenged animal did not express any endpoint criteria after 28 days. (**D**) Kaplan-Meier survival analysis between control group and high H3 group. Significant improvement in survival was observed between sonicated group and control group (*P* < 0.05). (**E**) Flow cytometry plot showing CD4^+^ and CD8^+^ lymphocytes (gated on CD45^+^CD11b^−^) in the rechallenged brain. (**F**) Flow cytometry plot showing CD69 and CD103 expression (gated on CD45^+^CD11b^−^CD8^+^) on CD8 T cells in the rechallenged brain (black) and blood (red). (**G**) Quantification of total CD69^+^CD103^+^CD8 T cells in the rechallenged brain and blood.

Last, to assess the effectiveness of controlled anti-PD1 delivery in GBM, we conducted survival studies between the control group (anti-PD1 only, *n* = 9) and high H3 group (anti-PD1^+^ controller, *n* = 7). At 7, 12, and 17 days after GL261 implantation, we sonicated five nonoverlapping areas (high H3 group) and injected anti-PD1 shortly after the completion of sonication. The control group (*n* = 9) was treated with only anti-PD1 on the same days (Fig. 5A). After the third treatment, the mice were monitored until they reached the endpoint criteria. We found that closed-loop controlled MB-FUS in combination with anti-PD1 conferred a small but statistically significant improvement in survival, as compared to anti-PD1–treated animals with no sonication (*P* < 0.05) (Fig. 5, C and D). One long-term survivor remained in the combined group (anti-PD1^+^ controller), where the tumor completely disappeared. After a rechallenge experiment, we found that the mouse had developed resistance to GL261 cells, suggesting that the treatment elicited antitumor immunity (Fig. 5D). Analysis of the immune infiltrate of the survivor’s brain and blood at 4 months after rechallenge indicated that this mouse had a notable presence of CD8^+^ T cells expressing tissue-resident (CD69^+^CD103^+^) markers within the brain (Fig. 5, E and F) (*52*, *53*). The blood was negative for these markers, indicating that minimal blood contamination was present and anti-PD1 therapy combined with MB-FUS induced sufficient long-lived memory T cell formation specifically within the brain. Last, postmortem analysis of the tumors in the mice that did not survive through immunostaining revealed no differences in the number of CD4 and CD8 T cells across the groups (fig. S8, A and B). Collectively, these findings allowed us to refine our understanding on the role of BBB/BTB in attenuating the therapeutic impact of immune checkpoint blockade in GL261 glioma tumors and demonstrate the abilities of closed-loop controlled MB-FUS to augment brain cancer immunotherapy when combined with immune checkpoint blockade.

## DISCUSSION

In this study, we presented a closed-loop system to control the cerebrovascular MB dynamics, characterized its performance and impact on the BBB/BTB phenotype, and assessed its ability to promote antitumor immunity when combined with immune checkpoint blockade (anti-PD1). The proposed controller can (i) attain and sustain close but distinct AE levels, (ii) respond to steep changes in MB concentration (MB uptake), (iii) track and adapt its performance to MB kinetics (uptake and clearance), (iv) incorporate safety states and respond to the onset of inertial cavitation (broadband emissions) to prevent tissue damage, (v) account for biological variability that characterizes brain tumors, and (vi) use more optimized cavitation threshold model from future sonications.

Over the past years, both open-loop (static control law) controllers (*54*–*57*) and closed-loop (dynamic control law) controllers (*42*, *58*–*63*) have been proposed for controlling the MB dynamics. Open-loop controllers use small and static preset input step size and closed-loop controllers use error term (P-control or PI-control) to calculate the input step size dynamically (*21*). While open-loop controllers provide stability, their rise time is limited by the small step size, so they lack the ability to respond to changes in MB concentration that as we showed is important to ensure safety (Fig. 1D). Meanwhile, in a closed-loop controller, the most commonly used control law is proportional control (P-control) (*59*, *61*, *64*) or proportional-integral control (PI-control) (*42*) because of its fast response to large gaps between current and target level. While P-control itself enables steady approach to a target state with a constant gain (*59*, *61*), some studies included additional steps to ensure stability and prevent divergence. These include (i) decreasing gain by 70% when target level is met (*42*), (ii) having an integral term to cease operation (*42*), (iii) modifying the gain or changing the input from pressure to pulse length depending on different controlling phases (*62*), and (iv) minimizing the gain that achieves predefined operation band based on accumulated datasets (historical) (*63*). However, optimizing all the steps and gains requires extensive experiments and may need to be done for each case separately (*64*). Our approach, where we adaptably modify the gain depending on the cavitation threshold model, enables optimization of the gain in real time while being capable of updating the cavitation threshold model through future/past sonication data to further improve its performance (fig. S3B).

In the context of closed-loop MB-FUS, the MB kinetics can significantly affect the safety profile of the controller (*21*). To account for it, previous studies either used slow MB infusion (*42*, *58*, *63*) and/or preset the controller’s operation time after MB administration (bolus) (*61*). While MB infusion mitigates some of the challenges associated with the fast MB clearance (less than 2 min), the need to spread the MB dose over longer time might render the detection of MB AE less sensitive (*21*). Moreover, such approaches do not account for biological viability, which can be exaggerated over the course of multiple treatments (i.e., multiple MB administration), for example, by anti–polyethylene glycol (PEG) immunity that can lead to accelerated blood clearance of PEGylated MBs, such as Definity (*41*). These effects, along with variations in MB size and dose (*65*), may explain the increasing pressure over the course of the sonication observed in recent investigations (*42*) and underscore the importance of real-time monitoring of the MB kinetics to ensure that the pressure levels remain below the threshold for inertial cavitation. On the other hand, the selection of fixed operation time limits the controller’s operation to about 17 s (2.5 times shorter than in our investigations) (*61*), which places a ceiling on controller’s maximum efficacy. This approach does not account either for biological viability, while to avoid divergence it requires to carefully time the administration of MB and the initiation of the controller. Our data demonstrated (Figs. 1 to 3) that the proposed controller can effectively track the MB kinetics and automatically adjust its output to ensure safe operation (fig. S4). Furthermore, our closed-loop algorithm is capable to accumulate input-output data to adapt the threshold levels to in situ changes in MB concentration (fig. S3E).

As we alluded to, our investigations also revealed that during conventional constant pressure MB-FUS, the likelihood of broadband occurrence during the initial MB arrival to the brain is particularly high (42% of total occurrences). This indicates that MB dynamics may be significantly affected not only by the vessel compliance but also by the interbubble dynamics. These findings demonstrate that without a controller, conventional MB-FUS is vulnerable to broadband emission during a sharp change in MB concentration and underscore the importance of controlled methods (i.e., gradual rise in excitation) for attaining safe and effective treatment. Our assessment on the adaptive aspects and performance of closed-loop controller highlights its robustness and its potential to be seamlessly integrated to current clinical systems (*22*). Because of its fast rise time, this controller can also be used to sonicate at optimal pressure multiple targets (through electronic beam steering) with a single MB administration, which is essential to cover larger areas and tumors (*22*), while remaining within the FDA limits of maximum permissible MB dose. The proposed controller can be combined with passive acoustic mapping (PAM) methods to further increase our capabilities to control the MB dynamics at high spatial and temporal resolution within or outside the brain (*60*, *66*, *67*). Different pulsing schemes for tracking the MB kinetics can also be envisioned and readily integrated to the proposed controller.

Our investigations also demonstrated that the proposed controller can (i) elicit specific changes in the BBB phenotype (AE-dependent increase in permeability and ICAM-1 expression) in healthy mice, (ii) enhance the delivery and penetration of anti-PD1 in GL261 gliomas, (iii) increase the interaction of macrophages with anti-PD1 in the GL261 TME without promoting global inflammation in nontumor tissue, (iv) improve the survival in GL261 tumor-bearing mice, and (v) synergize with anti-PD1 therapy to promote tissue-resident memory T cell formation. Consistent with the training data, DCE-MRI indicated that the proposed controller can be used to tune the BBB permeability in healthy mice brains. Our findings corroborate past investigations on the value of contrast-enhanced MRI in predicting the up-regulation in ICAM-1 expression in healthy brains (*25*, *45*); however, in the GL261 TME, our data show that the level of AE is a better predictor of ICAM-1 expression. The AE-dependent level of ICAM-1 expression in the brain TME is also contrary to the low ICAM-1 expression reported in melanoma tumor models by Curley *et al.* (*32*), suggesting that the properties of the tumor TME need to be factored in when assessing the impact of MB-FUS in the brain TME and underscore the importance of controlled methods to promote desirable changes and compare findings across laboratories and clinical systems.

The observed improvement in anti-PD1 delivery under MB-FUS corroborates bulk tissue measurements provided by Sabbagh *et al.* (*27*) and demonstrated that closed-loop MB-FUS can reduce the steep gradients in anti-PD1 distribution and improve its accumulation in the GL261 TME in an AE-dependent manner. In addition to the improved anti-PD1 delivery and penetration in GL261 tumors, including regions that are dense in macrophages/microglia (IBA-1 staining; Fig. 4A), we also demonstrated that the combined treatment increased the local interaction of macrophages with the anti-PD1^+^ TME at 6 hours after sonication. While these observations did not result in significant changes in bulk macrophage accumulation in the brain TME as indicated by flow cytometry, they indicate that MB-FUS is sufficient to modify the phenotype of existing macrophages as shown by an increase in the number of CD64^+^ cells. Our observation on local microglial activation with IBA-1 staining is also consistent with previous findings by Leinenga *et al.* (*33*) and Bathini *et al.* (*68*), where they found enhanced microglial activation after MB-FUS in Alzheimer’s mice model, indicating the robustness of this response and their potential for facilitating therapy across different diseases. Consistent with previous investigations, our analysis indicated that MB-FUS did not increase the cytotoxic immune cell accumulation in the GL261 TME (*32*), suggesting that inertial cavitation might be needed to induce responses that are more pronounced than the already inflamed GL261 TME. Despite the limited changes in immune cell trafficking observed at 12 hours after sonication, our investigations resulted in improved survival in the PD1-expressing GL261 TME when anti-PD1 is injected after controlled treatment and induces sufficient long-lived memory T cell formation specifically within the brain, underscoring the importance of localized delivery and penetration of immune checkpoint inhibitors in the GBM TME. Together, our investigations allowed us to establish a link between changes in the strength and type of MB oscillation, BBB/BTB phenotype, and penetration/distribution of immune adjuvants (anti-PD1), and their impact on immune cell trafficking and antitumor immunity against aggressive brain tumors such as GBM.

Collectively, our findings demonstrate our ability to define therapeutic windows to augment brain cancer immunotherapy with MB-FUS and immune checkpoint blockade. Expanding the characterization of the inflammatory responses performed in healthy and diseased brains (*25*, *32*, *33*, *45*, *68*) using AE-controlled MB-FUS in brain tumor immune microenvironment and for different tumor types and exposure settings will allow to further refine the treatment window (i.e., FUS exposure) to elicit distinct immuno-mechano-biological effects and promote effective therapeutic trafficking in the brain tumors. In addition, our findings help refine our understanding on the role of the BBB/BTB in attenuating the therapeutic impact of immune checkpoint blockade in gliomas and reinforce the notion of spatially targeted brain cancer immunotherapy. Integration of the proposed closed-loop control methods with more detailed OMICS analysis may allow us to better understand the role of the BBB/BTB in health and disease and reveal additional immuno-mechano-biological responses to further define and refine the MB-FUS treatment window to promote spatially targeted immunotherapy (*35*). Closed-loop controlled MB-FUS can also be readily integrated with neurosurgical interventions to develop refined treatment protocols (e.g., through targeting the tissue around the resected cavity) that may significantly affect clinical outcomes (*69*, *70*). Furthermore, the proposed closed-loop controller can be readily used within or outside brain to reprogram the immune cells within the TME through gene transfection strategies to facilitate T cell recruitment and elicit antitumor immunity (*35*, *71*, *72*). Beyond brain cancer immunotherapy and the potential of the proposed approach to target the tumor core and infiltrating margin, which currently remains inaccessible to therapy, the proposed system, due to its safety profile, can enable effective drug delivery strategies into the clinic for a range of neurological and neurodegenerative diseases (*23*, *73*).

## METHODS

### In vivo experiments

All animal procedures were performed according to the guidelines of the Public Health Policy on the Humane Care of Laboratory Animals and approved by the Institutional Animal Care and Use Committee of Georgia Institute of Technology. Eight- to 10-week-old female C57BL/6J mice (The Jackson Laboratory) were used in this study (*N* = 63 mice in total).

### USgFUS system

The system used in this study is a custom-built portable system composed of (i) a 3D-printed anesthesia mask, (ii) an ultrasound-guided FUS system, and (iii) a motorized 3D positioning system (Velmex) that the USgFUS system is mounted on (see fig. S2). The system has two distinct functionalities: (i) guiding the FUS transducer focus to the desired location in the brain using active imaging and (ii) monitoring and controlling the real-time MB dynamics with passive cavitation detection (PCD). Thus, the system operates in two different modes: imaging mode and therapeutic mode.

In the imaging mode, the system creates a 2D ultrasonic image by raster scanning (30 mm by 30 mm window) using the ultrasound imaging probe that is coaxially aligned with the FUS transducer. To guide the FUS transducer to a target location in the brain, we fuse the information from the US image formed (i.e., by raster scanning the head) onto the preoperative MR image. This happens as follows: From the 2D ultrasonic image, we (i) manually locate an eye locator in the image (i.e., a reflective 3D-printed plastic plate) and (ii) overlay this image with a line defined by the MR image using the location of eyes. Desired target coordinates in the sagittal direction of the brain are then determined relative to this reference line. After the relocation of the FUS transducer to such target coordinates, one pulse/echo scheme is used to align the US focus to the desired depth in the brain (relative to the skull), by determining US travel time to the skull with sound speed of 1540 m/s. Using BBB opening experiments in healthy rodents, we were able to assess the targeting accuracy of the system (targeted region in preoperative MRI versus region with MR contrast agent extravasation) and found that it is ±500 μm accurate in *XYZ* coordinates (see fig. S2). This targeting precision is lower than the FUS focal region full width at half maximum (FWHM), in any direction, and thus sufficient for our experiments.

In the therapeutic mode of the USgFUS system, the imaging probe is converted into passive mode to serve as a passive cavitation detector during BBB opening procedures. When in passive mode, the recorded signal (11 ms long) by the transducer is high pass–filtered (cutoff of 2 MHz) before feeding to a data acquisition system (model 5000D, Pico Technology) and analyzed using fast Fourier transform from the host computer. The FUS transducer (Sonic Concept) is driven by a sinusoidal signal (1.64 MHz; 10-ms pulse length; 1 Hz pulse repetition frequency (PRF)) generated by function generator (Picoscope, Pico Technology), which has been amplified by a 50-dB power amplifier (model 240L, Electronics & Innovation Ltd). The focal pressure of the FUS transducer in the water (free field) was determined using a calibrated hydrophone (2 mm model, Precision Acoustics).

All the radiofrequency data (i.e., signals) were filtered using a high-pass filter with a cutoff frequency of 2 MHz (i.e., above the fundamental frequency of the FUS transducer: 1.64 MHz). The frequency bin we used for measuring broadband was 3.64 ± 0.0003 MHz (2.22 f0), as this was in-between the second harmonic and ultraharmonic and closest to the maximum sensitivity of the imaging probe/PCD, which was at 3.5 MHz. In addition, we used 4.10 ± 0.0003 MHz (2.5 f0) for the second ultraharmonic frequency band and 4.92 ± 0.0003 MHz (3 f0) for the third harmonic frequency band. The third harmonic frequency (4.92 MHz) was chosen to be our input to the controller before the study to establish a balance between (i) the transmission loss of signal from the brain due to the skull, (ii) nonlinearity generated by reflection, and (iii) sensitivity of imaging probe (3.5 MHz) and sampling rate of our oscilloscope (15.6 MHz) and, last, (iv) due to its high correlation with *K*^trans^.

### Design of closed-loop AE controller for BBB opening

We designed the closed-loop controller to achieve a target AE level (*L^k^*_target_; *k* depicts *k*th harmonic) of a predefined observer state (*k* = 3 in this study). First, we established the relationship between the AE and pressure by rapidly sonicating a target region with pressure ranging from 0 to 350 kPa (Fig. 1E). We then fit these measurements onto a hyperbolic tangent function (Eq. 1) to determine each coefficient *a_i_* and finally to obtain a continuous cavitation threshold curve. When the controller is active, *L^k^*_model_ (the ideal AE level at pressure *P_n_*) is first computed by substituting *P* in Eq. 1 by *P_n_* (Eq. 2). Second, ε_n_ (the difference between model and measured AE level) is computed from Eq. 3, which is used to determine the adequate gain *K_p_* for feedback loop (Eq. 4). To be more specific, this pre-obtained relationship model *L*(*P*) is used as a real-time reference in the controller to quantify how much the controller should “trust” its current AE measurement. For example, if the difference between current measured AE level (*L_n_^k^*) and model AE level (*L^k^*_model_) is large, small *K_p_* will be computed, which results in small pressure increment to be used in the next time point. On the other hand, if the difference is small (i.e., the measurement agrees with the model), larger *K_p_* will be computed, which results in high pressure increment to be used in the next time point. Note that here, the proportional gain *K_p_* is dependent on the inverse of instantaneous slope of cavitation threshold curve (*dP/dL*). This value, the coefficient of hyperbolic tangent function in Eq. 4, is infinite when the instantaneous slope is 0. The hyperbolic tangent function, by its nature, reaches a plateauing region when input (pressure in our study) is increased (Fig. 1E), where slope becomes near 0. This may result in infinite *K_p_* and ultimately result in divergence. Thus, here, we set the value *dP*/*dL* to be maximized when concavity (second derivative) crosses 0, which limits *dP/dL* to the maximum slope of the model *L*(*P*). Here, calculation of *K_p_* follows the smooth hyperbolic tangent function control law defined in our previous study (*60*). Last, the proportional feedback loop is completed by computing the error term *e_n_* (Eq. 5). Pressure increment (Δ*P*) for therapeutic pressure (*P*) is then the product of *K_p_* and *e_n_*; the resulting pressure for next time point *P*_*n*+1_ is calculated with Eq. 7. Note that all the variables, excluding *K_p_*, without subscript *n* are either a constant or a time-independent value.

$$L(P)=a_{1}\tanh(a_{2}P+a_{3})+a_{4}$$ (1)

$$L_{model}^{k}=a_{1}\tanh(a_{2}P_{n}+a_{3})+a_{4}$$ (2)

$$\varepsilon_{n}=\frac{|L_{model}^{k}-L_{n}^{k}|}{L_{model}^{k}}$$ (3)

$$K_{p}={(\frac{dL}{dP}|}_{P=P_{n}}){}^{-1}\tanh[{\left(\varepsilon_{n}+0.99\right)}^{-7}]$$ (4)

$$e_{n}=L_{target}^{k}-L_{n}^{k}$$ (5)

$$\Delta P=K_{p}e_{n}$$ (6)

$$P_{n+1}=P_{n}+\Delta P$$ (7)

To remove nonlinear signals produced from sources other than MBs, background signal spectrum (obtained a priori without MBs, 11 ms of recorded length, *f_s_* = 10 MHz, using similar pressures used during MB administration) was logarithmically subtracted from MB AE spectrum (see fig. S1). Isolated signals are then multiplied by 20 to be converted into decibel scale. Thus, the decibel units describing AE level in this study (for constant pressure sonications) are referenced to the background signal spectrum at the frequency of interest. However, because the controller may use pressure levels down to decimals, accurate subtraction of background emission is not practical. For controller’s operations, decibel values were calculated by subtracting broadband noise levels instead.

The controller is also able to track cerebrovascular MB kinetics using the MB tracking pulse, *P*_tracker_. MB kinetics tracking allows effective time window for controller’s operation. To prevent controller’s divergence caused by cerebrovascular MB clearance, we used an MB tracking algorithm that monitors the MB kinetics after they are injected in the animals. The MB tracking pulse is a constant, low-pressure pulse (1.64 MHz, 10-ms pulse length, 1-Hz PRF, and 70-kPa peak negative pressure) that follows the preceding controlled pressure pulse. The pressure for this pulse was chosen by selecting a pressure that resulted in minimal effect on BBB permeability from controller training data (Fig. 1C). We have established criteria for controller’s operation: (i) When this MB tracking pulse detects a 10-dB rise (relative to background signal) in the third harmonic (H3) level, the controller is turned on; (ii) after the controller is turned on, the initial slope of MB clearance is monitored for 40 s and is updated in real time after the initial recording; and (iii) when the calculated slope indicates 20% decay from the normalized maximum H3 level, the controller is ceased, and therapeutic pulse is maintained at its latest calculated pressure. A brief schematic of the controller operation protocol can be found in fig. S4. The target levels that were used in the study were chosen to represent two of MB dynamics regime: weak stable cavitation and strong stable cavitation. Weak stable cavitation was characterized by mean H3 level of 26 dB (where less than 3% of ultraharmonic emissions occurred), and strong stable cavitation was characterized by mean H3 level of 30 dB (where less than 3% broadband emissions occurred).

### Experimental procedures

All sonications were performed with the FUS transducer operated at 1.64 MHz, with a 10-ms pulse length and a 1-Hz pulse repetition frequency, for 120 s under concurrent intravenous administration of clinical-grade MBs (100 μl/kg; Definity, Lantheus Medical Imaging). During the sonications, we recorded/controlled the AE using the single-element PCD. During BBB opening experiments in healthy mice for controller training, we sonicated four target regions in the brain (two in each hemisphere) with constant focal pressures. DCE-MRI was taken right after the sonication sessions. Similarly, for controlled BBB opening experiments in healthy mice, we sonicated four target regions in the brain (two in each hemisphere) using the real-time controller, which varied the focal pressure according to desired threshold values (see above), followed by DCE-MRI. During controlled BBB opening experiments in brain tumors, we performed five nonoverlapping sonications (*XY* directions, separated by 1 mm) to cover the entire tumor and its periphery. Shortly after the sonication, 100 mg/kg of control immunoglobulin G (IgG) (BP0089, Bio X Cell) or anti-PD1 antibody (BP0146, Bio X Cell) was injected intravenously.

To characterize the brain tumor microenvironment and assess inflammatory responses and anti-PD1 antibody delivery in healthy brain and GL261 tumors, the animals were euthanized at 6 hours after treatment. The mice were transcardially perfused with 20 ml of saline before harvesting the brains. Then, the brains were fixed with 4% paraformaldehyde overnight at 4°C, followed by 30% sucrose solution (4°C) until it sunk to the bottom of the container. The brains were placed in an optimal cutting temperature (OCT) compound and rapidly frozen to −80°C. Subsequently, 20-μm sections were cut using a cryostat (Leica 3050 S Cryostat).

### Dynamic contrast-enhanced MRI

To access the vessel permeability in the brain, we measured the volume transfer constant, *K*^trans^, by performing DCE-MRI (Pharmascan 7T, Bruker, IR; echo time, 2.5 ms; repetition time, 1019.6 ms; flip angle, 30; field of view, 40 mm by 40 mm). More specifically, we started collecting DCE-MRI with concurrent bolus administration of 8 μl of gadolinium contrast agent (0.4 ml/kg; Magnevist). The collected DCE-MRI datasets were analyzed, and *K*^trans^ values were calculated in OsiriX, using DCE tool plugin (Kyung Sung, Los Angeles, CA). The arterial input function was obtained on the basis of Fritz-Hansen *et al.* (*74*) method, as provided in the plugin.

### GL261 glioma cells and tumor inoculation

GL261 glioma cells (Caliper Life Sciences) were cultured in Dulbecco’s modified Eagle’s medium supplemented with 10% fetal bovine serum (FBS) and 1% penicillin-streptomycin at 37°C and 5% CO_2_. GL261 cells (10^5^ cells) were stereotactically implanted into the brain at 1 mm anterior and 1 mm to the right and 3 mm deep of the bregma of 6- to 8-week-old female C57BL/6J mice (The Jackson Laboratory). After cell implantation, tumor growth was monitored using T2-weighted MRI (echo time  = 35 ms, repetition time  = 2.5 s, rapid aquisition with refocusing echos factor = 8, slice thickness = 1 mm), and BBB opening was performed when tumors reached a size of ~20 to 40 mm^3^. To minimize differences related to tumor size, before each experiment, the tumors in all animals were measured with MRI and spread equally between treatment groups.

### Immunofluorescence staining and microscope imaging

Tissues were first prepared for staining by fixing in 4% paraformaldehyde at room temperature for 10 min. After washing tissues with phosphate-buffered saline (PBS), we blocked the sessions for 1 hour at room temperature (2% bovine serum albumin and 5% goat serum in PBS). We then incubated the tissues with primary antibody diluted in 1% bovine serum albumin (1:100) for 12 hours at 4°C. Next, the sections were incubated with secondary antibody diluted in 1% bovine serum albumin (1:250) for 1 hour at room temperature. To stain the cell nucleus, samples were incubated with 4′,6-diamidino-2-phenylindole (DAPI) diluted in PBS (1:1000) for 15 min after washing. Last, the sections were rinsed with PBS to remove excess antibody, mounted with mounting medium (ProLong Glass Antifade Mountant, lot no. 2018752, Invitrogen), and covered with coverslips. Samples were cured with a mounting medium for 24 hours in the dark at room temperature before imaging. The sections were imaged with a 20× objective using a fluorescence microscope (Eclipse Ti2, Nikon). The different stainings conducted along with information of the antibodies used are provided in Table 1.

**Table 1.** Summary of the antibody and microscope settings used for immunofluorescence imaging.

Target	Primary antibody	Secondary antibody	Microscope excitation
PD1	Rat anti-mouse PD1 (100 mg/kg delivered in vivo; BP0146, Bio X Cell)	Goat anti-rat Alexa Fluor 647 (A21247, Invitrogen)	639 nm
CD31	Rabbit anti-mouse CD31 (ab28364, Abcam Inc.)	Goat anti-rabbit Alexa Fluor 488 (A11008, Invitrogen)	488 nm
ICAM	Rat anti-mouse ICAM-1 (14-0542-85, Invitrogen)	Goat anti-rat Alexa Fluor 647 (A21247, Invitrogen)	639 nm
Macrophages/microglia	Rabbit anti-mouse Iba1 (019-19741, Fujifilm, Wako Chemicals)	Donkey anti-rabbit Alexa Fluor 555 (A31572, Invitrogen)	555 nm
DAPI	DAPI solution (62248, Invitrogen)		405 nm

To quantify the immunofluorescence data and particularly the ICAM expression, we identified vessels from CD31 staining using tubeness function (sigma = 2). We then binarily multiplied the obtained values to the original ICAM-stained image. The mean of ICAM intensities was calculated for a region of five 0.1 mm by 0.1 mm areas in each sample. To quantify the delivery of anti-PD1, the mean intensity with colocalization of IBA-1 staining was obtained for a region of five 0.1 mm by 0.1 mm areas in each sample. All image processing was done using ImageJ (*75*).

### Immunohistochemistry staining and microscope imaging

Tissues were first prepared for staining by fixing in 4% paraformaldehyde at room temperature for 10 min and then washed with PBS. After neutralization of the endogenous peroxidase with 3% H_2_O_2_ for 10 min, the sections were incubated with a protein blocking buffer for 10 min before undergoing incubation with the primary antibody. Anti-CD4 (14-0041-85, eBioscience) and anti-CD8 (14-0081-85, eBioscience) staining was developed using 3,3'-Diaminobenzidine (Vector Laboratories, Burlingame, CA), followed by hematoxylin counterstaining (MilliporeSigma, St. Louis, MO). Hematoxylin and eosin (H&E) staining was also performed to access the location of the tumor. Anti-CD4 and anti-CD8 cells were manually counted at five different 0.1 mm by 0.1 mm regions.

### Flow cytometry

At 12 hours after treatment, mice were euthanized and transcardially perfused with ice-cold PBS. Tumors were manually dissected from nontumor tissue and processed for flow cytometry along with the contralateral non–tumor-bearing brain hemisphere. Tissues were mechanically disrupted and incubated in type IV collagenase (150 U/ml; Worthington, LS004209) with deoxyribonuclease (Worthington, LS002007) for a total of 45 min at 37°C in 1× PBS. Tissue homogenates were then filtered through a 70-μm cell strainer, and the lymphocytes and other immune cells were purified by a 30 to 70% Percoll gradient (GE Healthcare, 17-0891-01) by centrifuging at 650 rcf at 20°C for 20 min. After collecting the leukocyte layer, cells were washed with PBS and plated in a 96-well U-bottom plate for staining. All antibodies for flow cytometry were purchased by BD Biosciences, BioLegend, Cell Signaling Technology, and R&D Systems. For cell surface staining, antibodies were added to PBS in 1:20 to 1:200 dilutions and applied to the cells for a 30-min incubation on ice. Cells were then washed two times with PBS supplemented with 2% FBS and 0.4% EDTA and then fixed with fixation/permeabilization solution (eBioscience, 00-5523-00) for 25 min at room temperature. Cells were then intracellularly stained for purified anti-TCF1 (Cell Signaling Technology, 2203S) diluted in permeabilization buffer. After two washes with permeabilization buffer, cells were then further stained with goat anti-rabbit IgG Alexa Fluor 488 (Invitrogen, A11070) for 30 min on ice. After all staining, cells were washed two times with PBS supplemented with 2% FBS and 0.4% EDTA and resuspended for flow cytometry. Flow cytometry was performed on a BD FACSymphony A3 analyzer and further analyzed in FlowJo.

### Survival study

Mice were treated with (i) anti-PD1 antibody (100 mg/kg for each treatment; BP0146, Bio X Cell; *n* = 9) and (ii) anti-PD1 antibody (100 mg/kg for each treatment; BP0146, Bio X Cell; *n* = 9) with FUS-BBB/BTB opening. The treatments are performed at days 7, 12, and 17 after tumor inoculation, as described above. Following the treatment, the tumors were imaged every 3 days with MRI. The animals were euthanized using the endpoint criteria (exhibited severely impaired activity, weight loss exceeding 20% within 1 week compared to the baseline before the treatment, or if treatment-related severe adverse events occurred that caused pain or distress and that could not be ameliorated). Of the nine animals, there were two animals that were excluded from MB-FUS (high H3) + PD1 group. One animal had severe tail damage after two treatments that hindered the intravenous injection, and the other died from treatment due to high dose of anesthesia. The animal that survived from anti-PD1 antibody with FUS-BBB/BTB opening group was rechallenged at 70 days after the first tumor inoculation with the same tumor inoculation procedure described above and imaged every 2 to 3 days with MRI.

### Statistical analysis

Results are expressed as means ± SEM. All statistical analyses were performed using MATLAB and GraphPad Prism. *P* < 0.05 was considered statistically significant. All animals were randomly and blindly distributed by color codes into control and treatment groups.

## Notes

View/request a protocol for this paper from *Bio-protocol*.
