# Supplementary material for: Spatially targeted brain cancer immunotherapy with closed-loop controlled focused ultrasound and immune checkpoint blockade
Source: Sci Adv. 2022 Nov 18;8(46):eadd2288. doi: 10.1126/sciadv.add2288 (PMC9674274; doi:10.1126/sciadv.add2288)
Supplement: Supplementary file 1 — Supplementary Materials and Methods Figs. S1 to S8 [file sciadv.add2288_sm.pdf]

Science Advances
AAAS

Supplementary Materials for

Spatially targeted brain cancer immunotherapy with closed-loop controlled focused ultrasound and immune checkpoint blockade

Hohyun Lee *et al.*

Corresponding author: Costas Arvanitis, costas.arvanitis@gatech.edu

Sci. Adv. **8**, eadd2288 (2022)
DOI: 10.1126/sciadv.add2288

This PDF file includes:

Supplementary Materials and Methods
Figs. S1 to S8

Supplementary Materials and Methods

1. Spectral bands employed in the controller

Passive cavitation detected frequency spectra are obtained using the imaging probe (**Fig. S1**). Before digitization, the data were high-pass filtered with 2 MHz cutoff frequency. Bin sizes are 59 Hz with sampling frequency of 15.6 MHz. The logarithmic levels, written with unit of Log.AU, of each of the frequency bands of interest (U2, H3, BB) are calculated by averaging the log of nearest 5 bins ($\pm$0.0003 MHz) of 1.5, 3, and 2.22 multiples of fundamental frequency (1.64 MHz), respectively.

2. Neuro-navigation method

After raster scanning, the eye locater (3D printed with PLA) that acts as a reflector, using pulse-echo ultrasound, the target coordinates in the brain are determined using T1 weighted MR image (**Fig. S2**). The two coordinates are then referenced to each other allowing to guide FUS transducer to target location. Contrast enhanced MR revealing the location of BBB opening in healthy mice after the application of MB-FUS are used to determine the targeting precision.

3. Adaptive learning and training of cavitation threshold model

After sonication, accumulated data of input (pressure) and corresponding output (H3) can be used to create a more trained cavitation threshold model that is used in controller's algorithm. Compared to the current model (**Fig. S3A**), which is obtained from one target on one animal, the model is a historic model that represents the MB dynamics from all sonications that was done by the controller. Healthy sonication (**Fig. S3B-D**; blue) did not differ significantly from tumor sonication (**Fig. S3B-D**; red), and thus is counted towards the overall training dataset. The vertical spread at certain pressure (**Fig. S3B**) suggests the decaying MB kinetics over time. Using the MB kinetics that were recorded at every sonication timepoints (**Fig. S3C**), and with its normalization (**Fig. S3D**), different models can be generated for each interval of MB concentrations; for example, **Fig. S3E** contains cavitation threshold model with 20% MB concentration inverval, and ideally, much finer intervals (e.g, 5%) can be generated accurately with more dataset.

4. Controller logic

For the uncontrolled (constant pressure) sonications used to train the controller, the FUS immediately begins sonicating at 0 s (**Fig. S4**, left). After acquiring background signals for 10 s, MB are administered, and the sonication continues for 2 more minutes. For controlled sonication, FUS pressure remains at 0 kPa until the 70 kPa MB kinetics tracking pulse detects a 10 dB jump in the 3rd harmonic (MB arrival). From this moment, the pressure starts to be controlled until the target level is met. The control continues until 20% MB decay and/or 2 minutes of sonication (**Fig. S4**, right).

5. Assessment of the safety features of the controller

The system was designed to suppress broadband emission throughout entire sonication, even when controller is not active (**Fig. S5**). This function, together with MB kinetics tracking function, which prevents divergence of controller, composes the safety feature of the system. To demonstrate the safety of the controller, we sonicated a new animal with the safety features of the controller active on the left hemisphere and inactive on the right hemisphere of the brain with a target H3 level of 36 dB. According to the training data (**Fig. 1C**), this level would result in

broadband emissions in more than 3% of the sonications. Using this level, we observed that the controller was able to react, in real-time, to broadband emissions and reduce the pressure (**Fig. S5A**; left, blue arrows). Likewise, the MB tracking prevented the pressure to diverge by ceasing the controller's operation after detecting 20% reduction of the MB emissions (i.e., MB concentration). On the other hand, when the safety features were absent, the controller was not able to react to broadband emissions. Importantly, divergence (continued increase in pressure) started to appear at approximately 25% MB clearance (**Fig. S5A**; right). During this time, probability of broadband occurrence was highest, further emphasizing the role of MB kinetics tracking that ceases controller's operation at optimal time point. As confirmed by H&E staining, the target with the safety features did not show any noticeable damage, while the target without them lead to significant tissue damage (i.e., petechiae formation; **Fig. S5B**). Collectively, these data clearly demonstrate that the proposed closed-loop controlled FUS system can sharply tune the exposure settings to safely promote controlled changes in the BBB permeability and expression of the key inflammatory marker ICAM-1 in healthy mice brains.

6. Analysis of immune cell trafficking in brain tumors 12 hours post FUS

To analyze the post-treatment inflammatory effects of FUS in combination with PD1 therapy, tumors and contralateral non-tumor tissue were processed for flow cytometry 12 hours after treatment to detect a variety of immune cell populations (**Fig. S6-7**). Results demonstrate an enrichment of all immune cell populations in tumor tissue compared to non-tumor tissue. Further, there were no statistically significant differences between treatment groups in tumor tissue for any immune cell population analyzed, indicating FUS either alone or in combination with anti-PD1 treatment does not induce any short-term inflammatory side effects in tumor tissue and in non-tumor tissue that received the same FUS treatment. Analysis of PD1+CD8+ T cells also demonstrates a notable presence of "stem-like" TCF1+TIM3- CD8 T cells and lack of "exhausted" CD101+TIM3+ CD8 T cells, suggesting that enhanced delivery of anti-PD1 antibody with MB-FUS may lead to a better thereapeutic response.

7. Postmortem analysis of CD4+/CD8+ cells in brain tumors

The postmortem samples after survival (excluding the long-term survivor) were IHC stained (**Fig. S8**). The positive cells were manually counted at five different locations in the microscopy images for each animal. There was no evidence of differences in CD4+ nor CD8+ cells between the control (PD-1) or treatment (FUS + PD-1) groups.

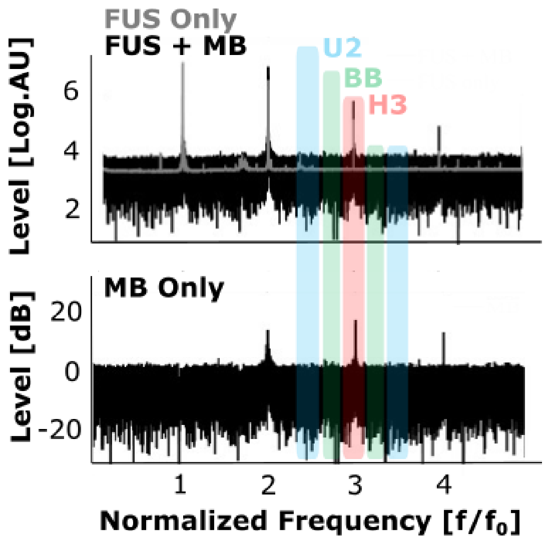

Fig. S1. The frequency bands that were analyzed for each 3rd harmonics, 2nd ultra-harmonics, and broadband emissions (bands not to scale).

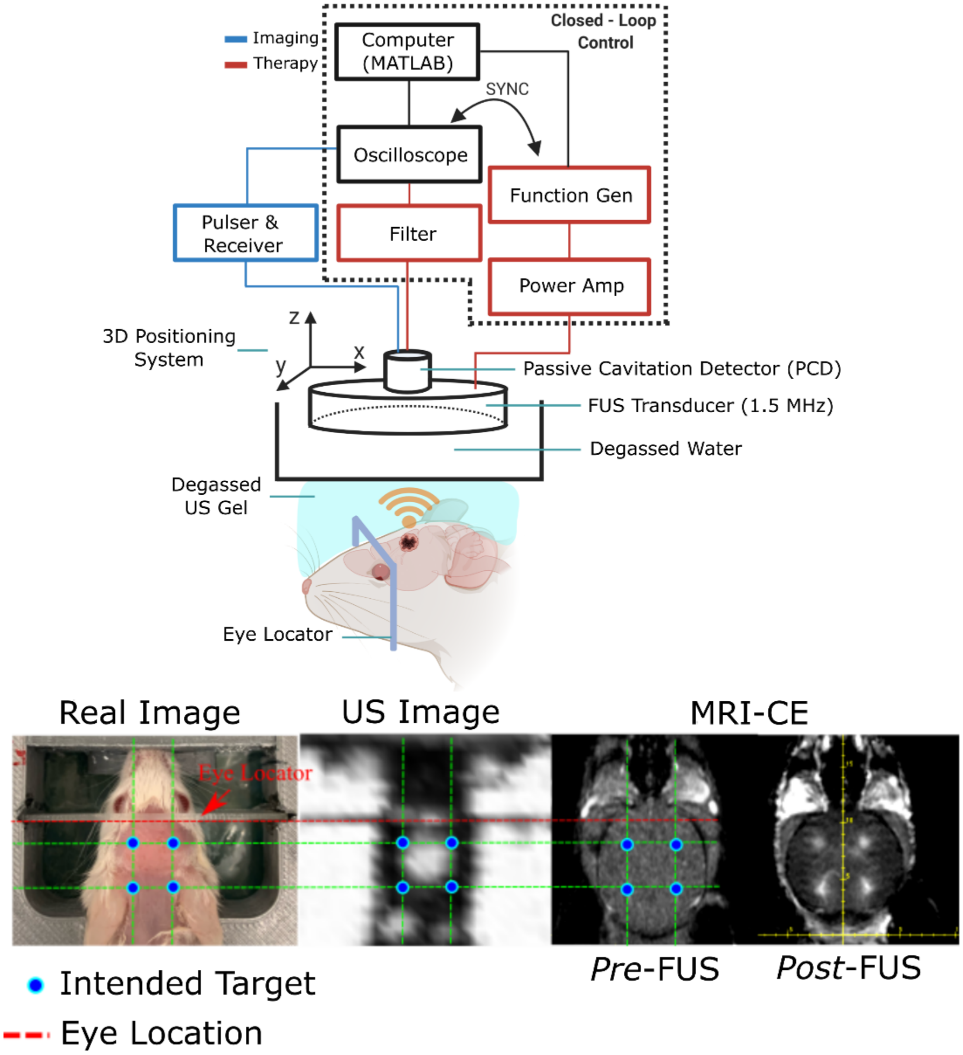

Fig. S2. Schematic the MB-FUs system, assessment of targeting precision using the eye locator

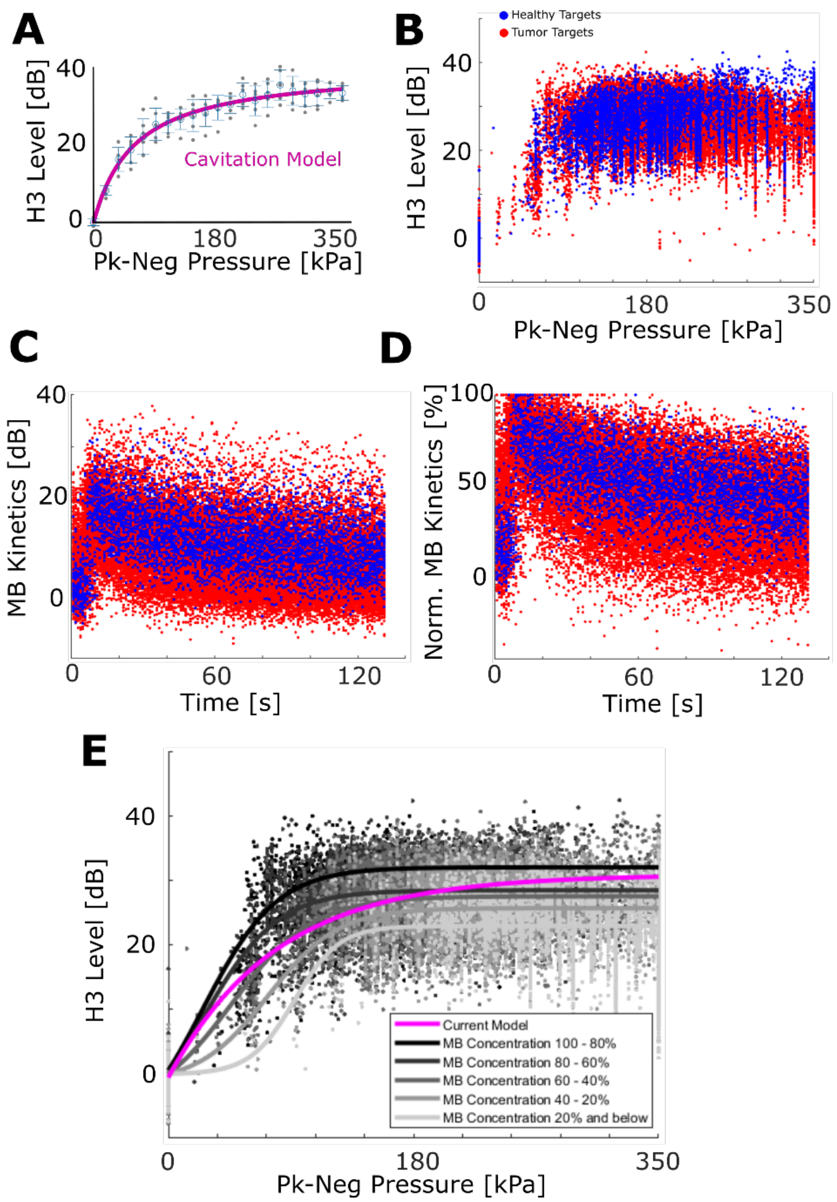

Fig. S3. Adaptive model learning of controller algorithm with accumulated data. A) Current model that was used in this study. Pressure sweep was recorded starting from 10 seconds after bolus injection of MB with 5 Hz PRF. B) Accumulated dataset obtained throughout study. Tumor sonication include approximately 60000 data points, and healthy sonications include approximately 5000 datapoints. C) MB kinetics for all sonication in this study and D) its normalization was done respect to the maximum H3 level. E) MB kinetics-accounted models. Normalized MB kinetics were logically multiplied to panel B) for each concentration interval. Each line represent 20% decaying interval (the lighter the color, the less the measured MB concentration in the brain).

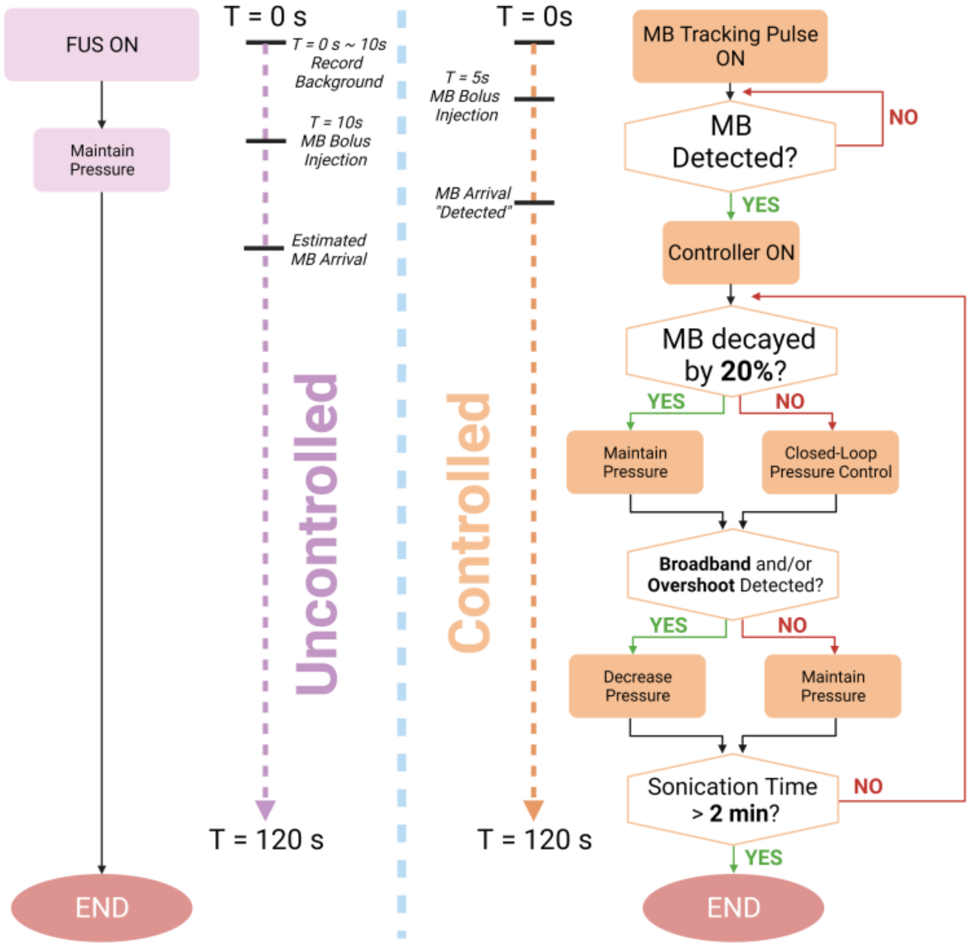

Fig. S4. The logical flow comparison between uncontrolled (constant pressure) MB-FUS and closed-loop controlled MB-FUS

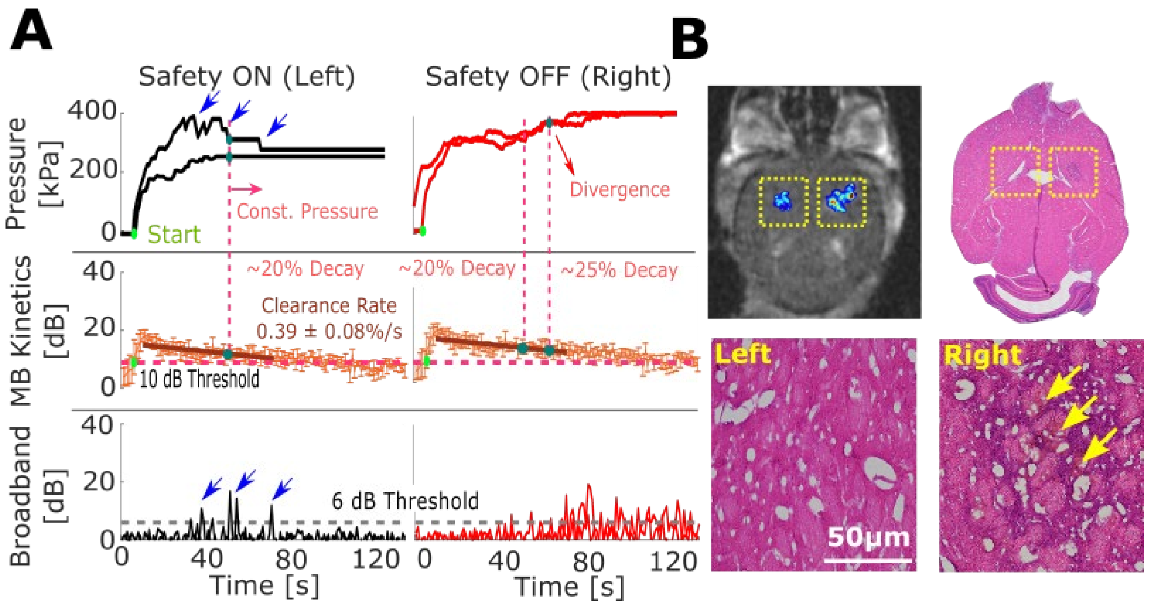

Fig. S5. Safety assessment of the controller with its safety functions (MB tracking and broadband emission detection) active and not active. A) With safety functions employed, controller was able to react to broadband emissions (blue arrows) even when the controller was ceased (right most blue arrow) and MB clearance at left target. Broadband occurrence with safety functions was 2.29% (6/262). Without safety functions, controller did not cope with broadband emissions as well as MB clearance, causing divergence at t ~ 60s at right target. Broadband occurrence without safety functions was 11.5% (30/262) B) H&E staining confirmed no damage at left target and petechiae formation (erythrocyte extravasation) at right target.

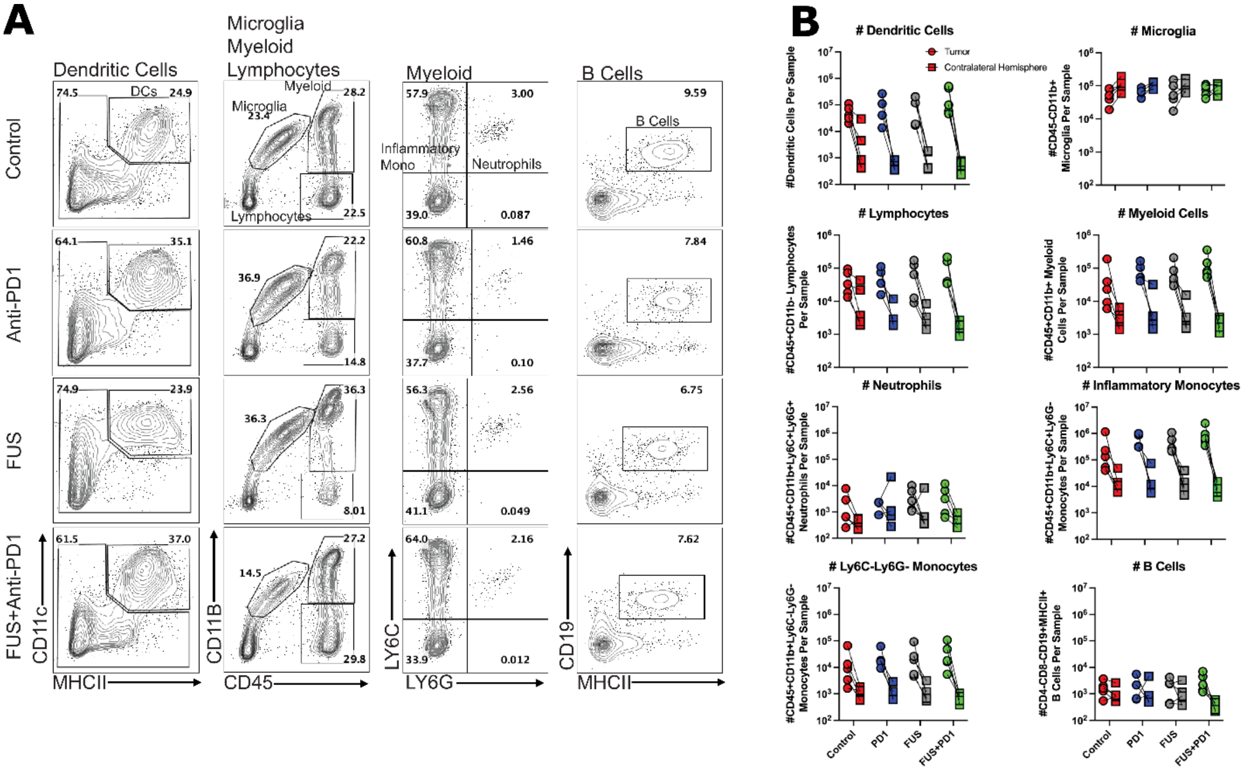

Fig. S6. Flow cytometry 12 hours after treatment. A) Representative flow cytometry plots of the indicated cell populations for each treatment group. Dendritic cells were gated on MHCII+CD11c+CD19- live cells. Myeloid, microglia, and lympchotye populations were gated off of their expression of CD45 and CD11b. Myeloid cells (CD45+CD11b+) were further gated on Ly6G and Ly6C expression to capture inflammatory monocytes (Ly6C+Ly6G-) and neutrophils (Ly6C+Ly6C+). B cells were defined as CD45+CD11b+CD4-CD8-MHCII+CD19+. B) Quantifications of the total cell numbers for each indicated cell population in the tumor tissue and contralateral non-tumor tissue.

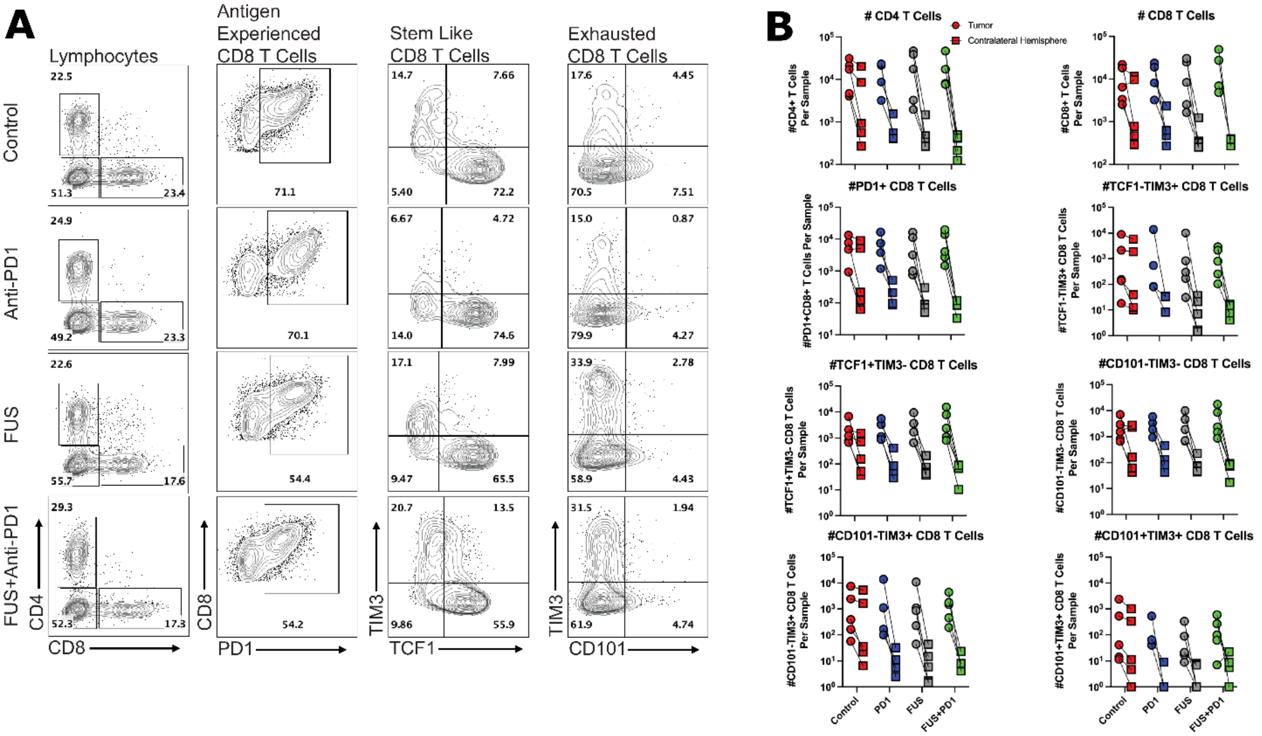

Fig. S7. Flow cytometry 12 hours after treatment. A) Representative flow cytometry plots of the indicated cell populations for each treatment group. Lymphocytes were gated on CD45+CD11b- cells. PD1+ CD8+ T cells were gated off of CD8+ T cells. Stem-like CD8 T cells and exhausted CD8 T cells were gated off of PD1+CD8+ T cells. B) Quantifications of the total cell numbers for each indicated cell population in the tumor tissue and contralateral non-tumor tissue.

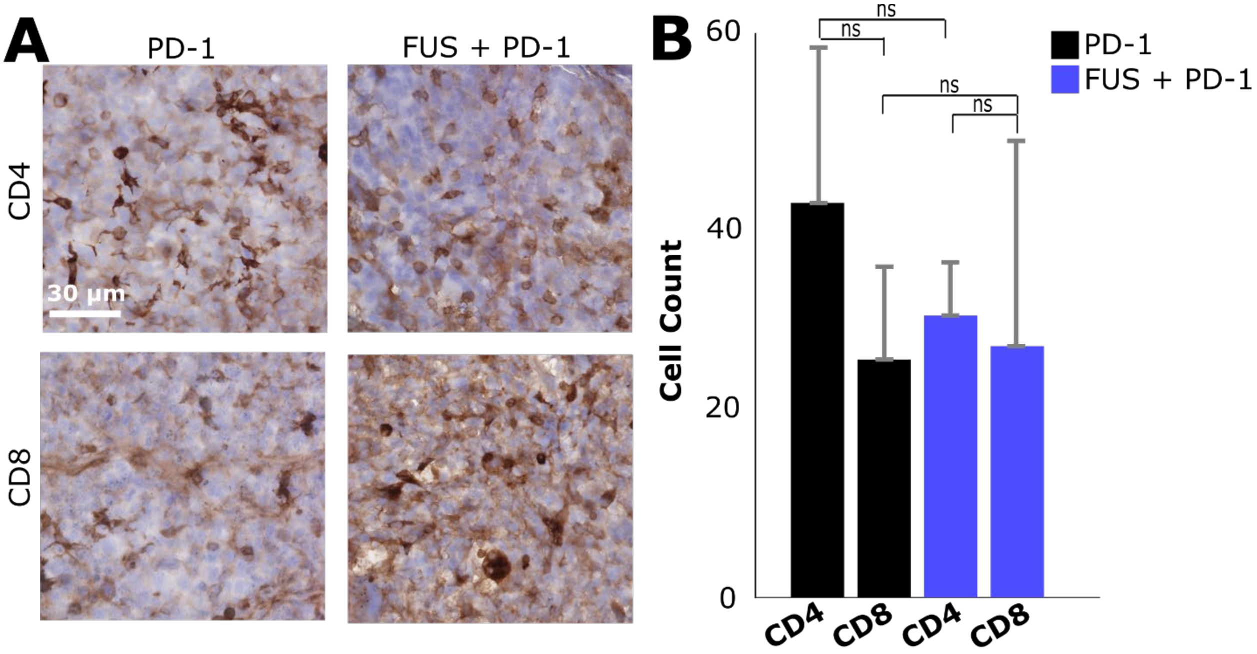

Fig. S8. A) Representative IHC microscopy data of CD4 and CD8. B) Quantification of CD4 and CD8. Number of CD4 and CD8 counts did not have any significance across the groups. One-way ANOVA was used as significant test; error bars indicate standard error.
